# Feasibility of Endoscopic Thyroidectomy via Axilla and Breast Approaches for Larger Goiters: Widening the Horizons

**DOI:** 10.1155/2018/4057542

**Published:** 2018-10-02

**Authors:** Goonj Johri, Gyan Chand, Nitish Gupta, Chaitra Sonthineni, Anjali Mishra, Gaurav Agarwal, Sabaretnam Mayilvaganan, Ashok Kumar Verma, Saroj Kanta Mishra

**Affiliations:** Department of Endocrine Surgery, Sanjay Gandhi Post Graduate Institute of Medical Sciences (SGPGIMS), Lucknow 226014, India

## Abstract

Scarless (in the neck) endoscopic thyroidectomy (SET) has evolved into a cosmetically preferred alternative to conventional thyroidectomy (ConT). Recently many of our patients are demanding SET; however their goitres are larger than the recommended size of 4–6 cm. Our aim was to compare the outcomes of ET for small (<6 cm) vs large (≥6 cm) goitres and determine its feasibility in such cases. This is a retrospective analysis of prospectively maintained database of patients undergoing ET. Patients were divided into 2 groups: I, small (<6 cm) and II, large goitres (≥6 cm). Their demographic and clinicopathological profiles, operation time, conversion and complication rates, and hospital stay were compared. 99 patients (101 procedures) were included: group I, 60 patients (61 procedures), and group II, 39 patients (40 procedures). Mean tumor size (± SD) was 4.4 ± 0.9 cm and 6.7 ± 1.1 cm in groups I and II, respectively. The groups were comparable with respect to demographic and clinical profile except for mean duration of goiter [30.1 ± 32.6 months (group I) vs 60.5 ± 102.4 months (group I), p = 0.03] and gland weight [21.5 ± 15.3 grams (group I) vs 62.3 ± 51.3 grams (group II), p = 0.001]. Although there was no significant difference between mean operating times, long term perioperative outcomes, and conversion rates, temporary hypocalcaemia and length of stay were longer in group II. One patient had permanent vocal cord palsy (~1%, 1/101); none had permanent hypoparathyroidism. Our results indicate that ET can be offered to a subset of patients with larger goitres desirous of SET with no significant difference in mean operation time, conversions, and long term postoperative complications in experienced hands.

## 1. Introduction

Over the past two decades, scarless (in the neck) endoscopic thyroidectomy (SET) has evolved into a cosmetically notable alternative to conventional thyroid surgery (ConT). Majority of patients with goiters comprise women who are often concerned about the cosmetic appearance and visibility of the neck scar. The preponderance of women patients has probably played a major role in the rapid emergence and acceptance of endoscopic thyroidectomy (ET). The recommended thyroid nodule (TN) size cut-off for ET based on published literature ranges from 4 to 6 cm. In most developing countries including India, patients present with long standing and relatively larger glands [1]. However, the demand for cosmetic procedures is stronger than ever in such countries due to social and cultural pressures and of late even patients presenting with large TN insist for SET [2]. In this study we wanted to compare our outcomes of ET in small (<6 cm) vs large (≥6 cm) goitres and determine the feasibility of ET in larger goiters.

## 2. Methods

This is a retrospective analysis of prospectively maintained database. From September 2012 to December 2017, patients who underwent ET in our department with minimum six months' postoperative follow-up were included. We take up such cases after discussing the pros and cons of ET in detail. Cases were divided in two groups: group I, patients with small TN (<6 cm), and group II, large TN (≥6 cm). Out of the various approaches described in literature, we have performed ET via axillary and breast approach (ABA), bilateral axillary breast approach (BABA), and transoral vestibular approach (TOVA). All surgeries included have been performed by the same surgeon. Initially only euthyroid patients with TN < 6 cm with cytology of Bethesda category II, III, or IV were attempted. However, as our experience increased, we attempted larger lesions, toxic glands, and low risk malignancies in patients who demanded ET. For the purpose of this study, patients who underwent ET using ABA and BABA were included. The demographic and clinicopathological profiles, approach of ET, operation time, and conversion and complication rates were compared.

### 2.1. Surgical Technique

All procedures were performed under general anesthesia through endotracheal intubation, using standard advanced laparoscopic instruments except for a custom-made metal tunneler which is required for raising the flaps. Since 2015, all cases have been performed using 3DHD (three-dimensional high definition) vision system (Viking System, Inc., Westborough, MA).

**Figure 1:**
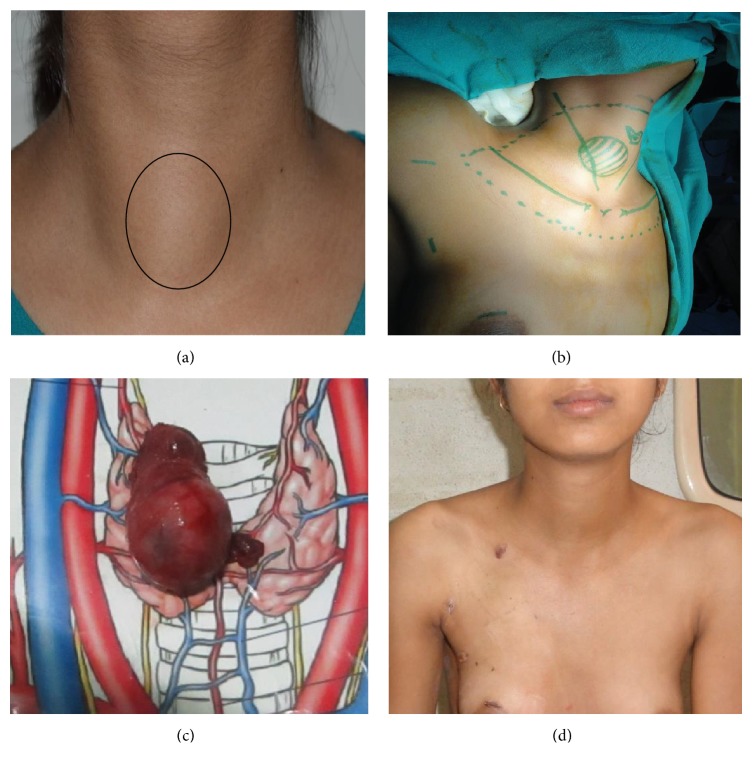
Axillo-breast approach (ABA): (clockwise from top left) (a) patient with right solitary thyroid nodule, (b) patient position and port placement, (c) specimen, and (d) postoperative outcomes.

**Figure 2:**
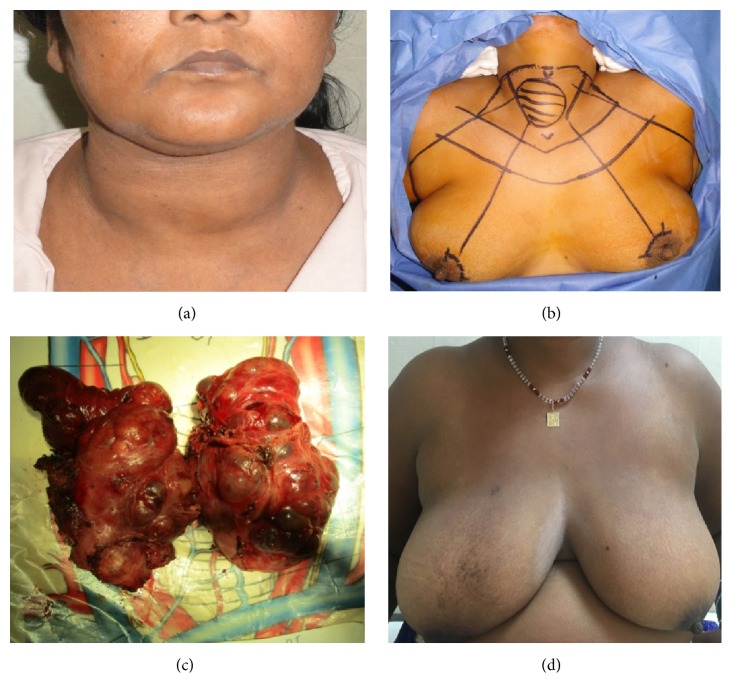
Bilateral axillo-breast approach (BABA): (clockwise from top left) (a) patient with MNG, (b) patient position and port placement, (c) specimen, and (d) postoperative outcomes.

**Figure 3:**
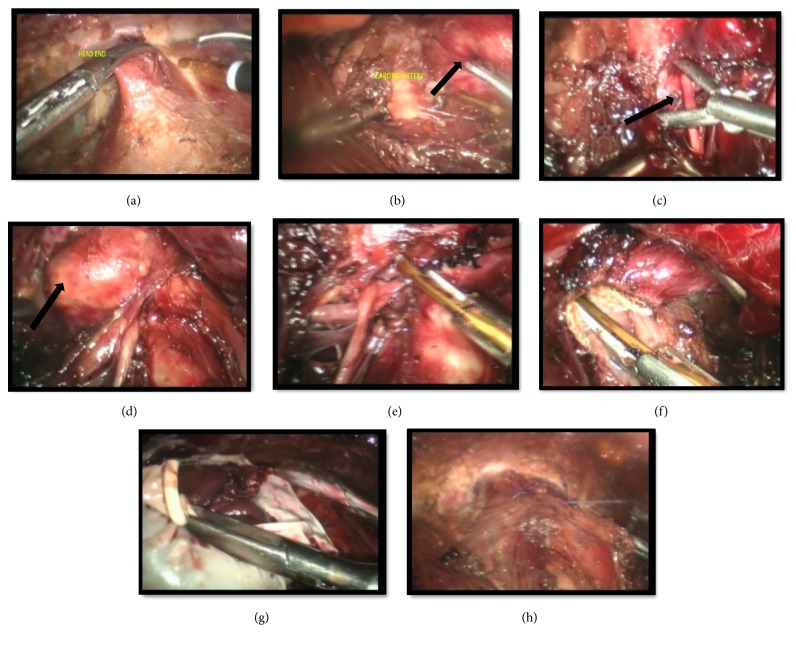
(a-h) Intraoperative view of key steps during endoscopic thyroidectomy. (a) Opening of cervical fascia in midline using ultrasonic hook. (b) Lateral dissection and identification of common carotid artery; medial extent is identification of trachea (not shown in image). Thyroid lobe is rotated medially (shown by black arrow). (c) Identification of RLN and (d) inferior PTH gland (both shown by black arrows). (e) Inferior vascular pedicle secured after safeguarding RLN and PTH (Ligaclip can be used to avoid thermal injury). (f) Isthmusectomy. (g) Specimen placed in surgical glove which works as a retrieval bag. (h) Midline approximated using polyamide 2-0 suture continuous stitch.

Patient was positioned supine with 30 degrees reverse of Trendelenburg's position, both arms adducted and partially flexed at the elbows. A sandbag placed vertically under the shoulders opens up the chest and aids in neck extension (Figures 1(b) and 2(b)). Chlorhexidine and povidone iodine were used for sterile preparation of neck and upper chest. A diluted solution of epinephrine with normal saline (1:200,000) was infiltrated into the subcutaneous plane over the chest and subplatysmal plane in the neck which helped in hydrodissection and hemostasis. After blunt dissection using tunneler over the chest and neck, ports were placed. We maintained insufflation using CO2 at 7mmHg with flow rate of 7 L/min. The flaps were further extended using ultrasonic energy device (Sonosurg, Olympus, Japan) from the level of hyoid superiorly to sternal notch inferiorly and laterally medial border of sternocleidomastoid on either side. Intraoperative views of key steps are shown in Figures 3(a)–3(h). Similar to ConT, midline was opened and strap muscles were separated from the visceral surface of the gland (Figure 3(a)). Next step was lateral dissection and separation from sternocleidomastoid. Identification of the carotid artery marks the limit of the same (Figure 3(b)). Middle thyroid vein was secured. A snake-shaped retractor was used to retract the strap muscles laterally.

In both ABA and BABA, the inferior vascular pedicle was secured first along with identification of the recurrent laryngeal nerve (RLN) and inferior parathyroid gland (PTH) (Figures 3(c) and 3(d)). Inferior thyroid artery was divided (Figure 3(e)) after identifying the RLN. For smaller nodules, isthmus was divided early on in the surgery to facilitate dissection; however in larger nodules it was divided later after securing the inferior thyroid vessels (Figure 3(f)). Thyroid lobe was then rotated medially and dissected off the trachea and finally pulled inferiorly to secure the superior pole and PTH. The specimen was placed in a surgical glove which served as an economical retrieval bag (Figure 3(g)) and was extracted by enlarging the 10 mm axillary port. Midline was approximated using polyamide 2-0 suture (Figure 3(h)) and a negative pressure drain (Jackson-Pratt) placed in the thyroid bed, all port sites were closed with absorbable sutures, and pressure dressing was applied. Policy was to keep the neck drain until output over 24 hours was 20 ml or less.

Postoperative care was the same as for conventional thyroid surgery.

### 2.2. Statistical Analysis

We used descriptive statistics for demographic and clinicopathological characteristics. To compare the means between the two groups, Independent-Samples* t*-test was used. To test the association between categorical data, Pearson's Chi square or Fischer exact test was used as appropriate. A p value <0.05 was considered as statistically significant. Statistical package for social sciences (SPSS) version 18.0 (IBM, Chicago, IL, USA) was used to analyse the data.

## 3. Results

Ninety-nine patients (101 procedures) were included. Group I comprised 60 patients (61 procedures) and Group II 39 patients (40 procedures). Mean age was 34.1 ± 11.5 years. There were 84 women and 15 men (F: M = 5.6:1). Mean tumor size was 5.3 ± 1.5 cm (range: 2.5-11) and mean duration of symptoms was 41.6 ± 69.5 months (range: 1- 480; median: 24 months). Mean serum TSH was 2.5 ± 2.2 mIU/L. Five patients were hyperthyroid and 6 had subclinical hypothyroidism. Total of 31 total thyroidectomies (TT), 63 hemi-thyroidectomies (HT), 4 completion thyroidectomies (CT), 2 TT with central neck dissection (TT+CCND) for papillary thyroid carcinoma (PTC), and 1 TT with CCND with unilateral selective neck (TT+CCND+SND) were performed. Details of demographic profile of patients and procedures performed in both groups are given in Table 1.

The two groups were comparable with respect to baseline demographic and clinicopathological characteristics except for mean duration of goiter which was significantly longer in group II [30.1 ± 32.6 months (group I) vs 60.5 ± 102.4 months (group II), p = 0.03]. This could possibly be the reason for larger gland size. In group I, one patient with AFTN (4.5 cm) underwent hemithyroidectomy whereas five patients with hyperthyroidism (4 toxic multinodular goiters and 1 case of Graves' disease) in group II successfully underwent endoscopic TT using BABA approach.

Details of procedures performed, mean operation time, and perioperative outcomes between the groups are shown in Table 2. The ex vivo mean gland weight was also higher in group II as expected [21.5 ± 15.3 grams (group I) vs 62.3 ± 51.3 grams (group II), p = 0.001). The mean postoperative length of stay (LOS) was significantly longer in group II [3.4 ± 1.4 days (I) vs 4.1 ± 1.2 (II), p = 0.01] which can be attributed to prolonged drain output in these patients secondary to larger area of dissection and wound cavity after resection. Although there was a higher incidence of transient hypocalcaemia in group II [38.9% (I) vs 58.2% (II)], it was not statistically significant. There was no significant difference in mean operation time, conversion rates, and perioperative and long term complications between the groups.

One patient in group I had permanent vocal cord palsy (0.99%, 1/101). No patient in either group had prolonged or permanent hypocalcaemia. There were no cases of tracheal or esophageal injury, postoperative hemorrhage, or wound infection. Overall conversion rate was 3.9% [n = 4/101, 1 in group I and 3 in group II (p value = 0.13)]. The reason for conversion was due to inability to secure primary hemorrhage from strap muscles for the one patient in group I and in group II due to intraoperative suspicion of malignancy (extra thyroidal invasion of strap muscle and trachea) in 2 patients and technical difficulty in accessing superior pole due to large nodule size in 1 patient.

Preoperative FNAC and final histopathology are given in Table 3. There were 11 patients with final histology reported as differentiated thyroid cancer (DTC), out of which 8 were Bethesda II, III, and IV on FNAC and 2 patients were Bethesda V and 1 patient was Bethesda VI on FNAC.

## 4. Discussion

Endoscopic thyroidectomy was first introduced by Gagner [3] in 1996 and Huscher [4] in 1997, who reported successful endoscopic parathyroidectomy and thyroid lobectomy, respectively. Micolli [5] in 1999 described the minimally invasive video-assisted thyroidectomy (MIVAT). Since then, over the past 20 years, numerous minimal access approaches have been described by various authors. According to the site of port placement, these can be broadly classified into cervical or minimal neck incision thyroidectomy (MNIT) and extracervical scarless (in the neck) thyroidectomy (ESNT) [6]. In order to avoid visible scar in the neck, extracervical approaches are most popular. Based on the access these are the anterior chest wall [7], axillary [8], the breast [9], and hybrid approaches such as the axillary-bilateral breast approach [10], bilateral axillo-breast approach [11], and the postauricular and axillary approach [12]. All these techniques have their own advantages and pitfalls.

As is the case with any new surgical technique, there is lack of sufficient clinical research and evidence. Even criteria for indications and contraindications are continuously evolving based on individual center experience [13]. However, the safety, feasibility, and cosmetic superiority of ET are well established now based on the work of experts all over the world [10, 14].

The largest series on ET of 512 cases by Choe et al, who developed the BABA approach and also laid out the inclusion criteria, reported a mean tumor size of 3.09 ± 1.49 cm in patients with benign nodules [14]. Most of our patients present with long standing goitres and larger nodule sizes as compared to most published literature. In the current study too, mean tumor size in group I is 4.4 ± 0.9 cm which is greater than that described in most series [9, 13–15].

Our hospital is a tertiary referral center in North India and we have been performing ET in our department since 2012. Initially we selected patients based on the published literature by experts in the field [10, 14, 15]. As the awareness of this procedure increased amongst the general population, more and more patients started demanding this procedure. Endoscopic thyroidectomy was considered only for patients who were desirous of scarless in the neck procedure and concerned about a visible scar. Clinicopathological parameters of such patients were evaluated to determine whether they fulfilled the inclusion criteria for SET.

In our initial days, only ABA was practiced by the operating surgeon (OS) with strict adherence to size criteria, functionality, and cytology, i.e., euthyroid glands, tumor size <6 cm, and benign cytology but the surgeon felt it was difficult to attempt larger nodules. Similar is the experience of others. In ABA and transaxillary approaches, the thyroid gland is approached laterally, working space is narrow, and consequently the instruments interfere with each other and contralateral lobectomy is not effectively accomplished as the contralateral RLN is not visualized properly [14].

BABA on the other hand is essentially a simulation of ConT and perhaps the most ergonomic of all described approaches. As our operative experience increased and after having received additional training in BABA, larger goitres, toxic glands, and low grade malignancies were attempted successfully. Currently BABA is the most commonly performed procedure as the authors feel that it is most feasible for moderate size and larger glands. It has some clear advantages like a symmetrical view, which allows surgery on both lobes; working ports in both hands allow ease in dissection of vital structures and control of hemorrhage, especially while operating larger/toxic glands. This was complimented with the use of 3D endoscopy which further aided us in precise dissection by enhanced depth perception. For smaller euthyroid benign nodules (<4 cm), we use natural orifice transoral vestibular approach (TOVA).

A recent series from India by Puntambaker et al [16] describes the experience of 3D endoscopy in 10 patients with nodules greater than 6 cm (range: 6-8.5) and concluded that 3D endoscopy helped them in managing such large tumors and ensuring safer surgery owing to greater depth perception and magnification. Another series of 3 patients by Duncan et al describes ET for TN ranging from 7.5 to 10 cm, using transaxillary approach. However, we did not come across any large series which compares the outcomes and feasibility of ET in small vs large goitres.

Cao et al. reported a mean operation time of 79.9 ± 20.1 mins for lobectomy and 89.9 ± 14.6 mins for TT. Choi et al. reported 151.2 ±38.1 min (range: 75–285 min) for TT and 141.7 ± 50.1 min (range: 84–360 min) for subtotal thyroidectomy or lobectomy. Our overall operation time is longer as compared to the other publications [10, 14, 16], although mean operation time between the 2 groups was comparable (p value >0.05 for HT, TT, and CT). This may be due to the learning curve of the surgical team per se, logistic reasons, such as preparation of instruments, and troubleshooting of apparatus which is very common in resource crunch setups like ours. This could possibly be overcome by accumulation of operative experience, good surgical view, and better instruments [13]. It took longer to operate on patients with Graves' disease and toxic goitres; however, the postoperative LOS and complications were similar. After the initial 50 cases and availability of 3D endoscopy (since 2015), our average operation time has reduced significantly.

The common complications encountered are similar to ConT. Complications unique to ET are superficial skin bruising, subcutaneous emphysema, chest wall paresthesia, and seroma [14, 17]. The reported incidence of transient and permanent hypocalcaemia after conventional thyroidectomy ranges from 0.3% to 49% and 0% to 13%, respectively [18]. The incidence of transient and permanent RLN palsy ranges from 0% to 6%, and ≤1%, respectively [18]. Choe et al. reported an incidence of 20.3% for transient and 1.7% for permanent RLN palsy. In our study, the overall rate of transient and permanent RLN palsy was 6% (6/101) and 0.99% (1/101), respectively [4 (6.5%) patients in group I and 2 patients (1.6%) in group II].

Most studies report an incidence of 0-30% and 0-4% for transient and permanent hypocalcaemia, respectively [14, 19, 20]. Our overall incidence of transient hypocalcaemia was 38.9% in group I and 52.4 % in group II. All patients recovered within 4-6 weeks. None of the patients in either group had permanent hypoparathyroidism.

The incidence of paresthesia over chest wall, skin bruising, and seroma was comparable within the two groups. Most patients reported mild to moderate paresthesia which subsided over a period of 1-3 months.

The reported conversion rates of ET in most reported series are 0-13% [10, 14, 17, 21, 22]. This directly relates to the prior endoscopic experience of the main surgeon with formal training as well as their own experience with conventional lobectomies [17]. Conversion rates amongst the two groups were comparable (p = 0.31).

The cosmetic results of both ABA and BABA ET are excellent [10, 14]. The operative scars are almost invisible after 3-6 months and most patients were extremely satisfied with scars and final cosmetic outcomes (Figures 1 and 2). Yeung et al. highlighted the regional variation in perception towards scars and how western women do not prefer scars around the nipple and favour the transaxillary approach [23]. For our patient population, especially young unmarried girls and their guardians, the matter of concern is any visible scar especially the neck and hence preference for ET via ABA/BABA. Recently there has been an increase in demand for natural orifice ET; however, the nodule size criterion for transoral vestibular approach (TOVA) is more stringent than all other approaches. Although a breast incision is inevitable in BABA endoscopic thyroidectomy, the circumareolar wound heals very well and is invisible from a distance (Figures 1(d) and 2(d)) [14].

To the best of our knowledge, this is the largest series comparing outcomes of SET in small vs large goitres and the data has been analysed from prospectively maintained records with adequate patient follow-up. However, the study has its limitations in that it is a single center experience with limited resources catering to a specific patient population with varied societal obligations and thus our results may not be generalizable to all setups. Although a formal cost-benefit analysis has not been done, the overall cost of ET is one and a half times more than that for Con T in our hospital.

## 5. Conclusion

We conclude that SET is a feasible option and can be extended to a wider subset of suitable patients with larger goitres who are desirous of ET with no significant difference in operation time, conversion rates, and perioperative and long term complications in trained hands. In our experience, bilateral axillary breast approach is the more suitable technique for large nodules and toxic glands.

## Figures and Tables

**Table 1:** Details of clinical presentation and demographic profile of patients.

**S. No. **	**Parameter **	**Group I (n=60)**	**Group II (n=39)**	**P value**
1	Mean age (years)	33.7 ± 11.3	34.9 ± 12.0	0.61

2	Gender ratio F:M	50:8 (6.3:1)	32:7 (4.6:1)	0.28

3	Mean tumor size (cm)	4.4 ± 0.9	6.7 ± 1.1	0.001*∗*
	Range (cm)	2.5-5.8	6-11	

4	Duration of goitre (months)	30.1 ± 32.6	60.5 ± 102.4	0.03*∗*
	Range (months)	1-144	2-480	

5	Clinical diagnosis	**(n=61)**	**(n=40)**	
	STN	46	19	
	MNG-both lobes	11	14	
	MNG-one lobe	1	1	
	AFTN	1	0	
	Graves' disease	0	1	
	Toxic MNG	0	4	
	Postoperative histology of differentiated thyroid carcinoma (DTC)	2	1	

7	Preoperative FNAC			
	Not done	3	1	
	Bethesda I	1	1	
	Bethesda II	44	30	
	Bethesda III	3	3	
	Bethesda IV	6	4	
	Bethesda V	1	1	
	Bethesda VI	3	0	

**Table 2:** Details of operative procedure, operation time, and perioperative outcomes.

**S. No. **	**Parameter **	**Group I (n=61)**	**Group II (n=40)**	**P value**
1	Approach - ABA	30 (49.2%)	9 (22.5%)	0.07
	- BABA	31 (50.8%)	31 (77.5%)	

2	Surgery performed			
	TT	12	19	
	HT	43	20	
	CT	3	1	
	TT+CCND	2	0	
	TT+CCND+SLND	1	0	

3	Conversion to open	1	3	0.13

4	Mean gland weight (grams)	21.5 ± 15.3	62.3 ± 51.3	0.001*∗*
	Range (grams)	6-60	18-253	

5	Mean Operation time			
	Hemithyroidectomy (mins)	152.0 ± 38.6	184.3 ± 85.5	0.15
	Total Thyroidectomy (mins)	206.4 ± 62.0	243 ± 57.92	0.22
	TT+CCND	220 + 28.3	-	
	Completion Thyroidectomy (mins)	96.5 ± 23.3	145	0.33

6	Mean hospital stay (days)	3.4 ± 1.4	4.1 ± 1.2	0.01*∗*

7	Complications			
	Transient RLN palsy	4 (6.5%)	2 (5%)	0.11
	Transient hypocalcaemia^¥^ (Biochemical/Clinical)	7/18(38.9%)	11/20(55.0%)	0.51
	Permanent RLN palsy	1(1.6%)	0	1.0
	Permanent hypoparathyroidism^¥^	0	0	
	Seroma	2.1 ± 0.1	2.0	1.0
	Skin bruising/hematoma	2.3 ± 2.6	2.0 ±	1.0
	Paraesthesia	2.1 ± 0.2	2.0 ±	0.51

*∗*: p value <0.05 was considered significant.

¥: transient hypocalcaemia and permanent hypoparathyroidism were calculated only for patients who underwent CT, TT, or TT+CCND±SLND.

**Table 3:** Details of final histopathology.

**Final Histopathology**	**Group I (n=61)**	**Group II (n=40)**
Benign (Colloid/MNG/Follicular adenoma)	50	30
Lymphocytic thyroiditis	2	3
Classical PTC	3	1
FVPTC	1	1
MIFTC	1	2
WIFTC	1	0
HCC	0	1
Primary hyperplasia	0	1
No evidence of malignancy	3	1

MNG: multinodular goitre; PTC: papillary thyroid carcinoma; FVPTC: follicular variant of PTC; MIFTC: minimally invasive follicular thyroid carcinoma; WIFTC: widely invasive FTC; HCC: Hurthle cell carcinoma.

## Data Availability

The data used to support the findings of this study are available from the corresponding author upon request.
